# A systematic review and meta-analysis of the association between uric acid levels and chronic kidney disease

**DOI:** 10.1038/s41598-022-10118-x

**Published:** 2022-04-15

**Authors:** Danilo Lemes Naves Gonçalves, Tiago Ricardo Moreira, Luciana Saraiva da Silva

**Affiliations:** 1 School of Medicine, Federal University of Uberlândia, Uberlândia, Minas Gerais Brazil; 2 Department of Medicine and Nursing, Federal University of Viçosa, Viçosa, Minas Gerais Brazil

**Keywords:** Chronic kidney disease, Chronic kidney disease

## Abstract

The function of uric acid (UA) in the genesis and evolution of chronic kidney disease (CKD) has motivated numerous studies, but the results remain inconclusive. We sought to conduct a systematic review and meta-analysis of cohort studies aiming to analyze the association of UA levels with the incidence and progression of CKD. Pubmed/Medline, Lilacs/Bireme and Web of Science were searched to identify eligible studies, following the PRISMA protocol. Data were presented for CKD incidence and progression separately. For the meta-analysis, studies with data stratified by subgroups according to serum UA levels were selected. The inverse variance-weighted random effects model was used to generate a combined effect estimate. Meta-regressions were performed to identify the causes of heterogeneity. The Newcastle–Ottawa Scale was used to assess the risk of bias. The publication bias was tested by funnel plot and Egger’s test. Eighteen CKD incidence studies (n = 398,663) and six CKD progression studies (n = 13,575) were included. An inverse relationship was observed between UA levels and protection from CKD incidence and progression. Lower UA levels were protective for the risk of CKD incidence (RR 0.65 [95% CI 0.56–0.75]) and progression (RR 0.55 [95% CI 0.44–0.68]). UA seems to be implicated both in the genesis of CKD and its evolution.

## Introduction

Chronic kidney disease (CKD) is a global public health problem affecting more than 697 million people^1^. The prevalence of CKD has been increasing worldwide, with an annual increase of 8 to 16%, which is higher than the general population growth^2,3^.

In recent decades, the function of uric acid (UA) in the genesis and evolution of CKD has motivated numerous studies, but the results remain inconclusive due to the complex and bidirectional interaction between the change in UA levels and renal function, which hinders the isolation of the causal effect of UA in the progression of CKD^4–6^. The coexistence of risk factors, such as hypertension and chronic inflammation^7–9^ and pathophysiological peculiarities of CKD make it complex to define the role of UA.

UA is the final product of purine catabolism from exogenous sources, through diet, and endogenous, by cell degradation^10^. The remnant of circulating UA accounts for more than half of the antioxidant potential of human blood^11^. However, when it is inside the cells, it exhibits a pro-oxidant behavior, stimulating the production of free radicals and pro-inflammatory cytokines, reducing the bioavailability of vasodilator substances and increasing vasoconstrictor substances such as angiotensin, which lead to oxidative stress, chronic inflammation and endothelial dysfunction, the tripod of the pathogenesis and progression of CKD^12,13^.

The growing interest in UA considerably increased the number of publications^14^, and some prospective observational studies^15–17^ and retrospective studies^18–20^ showed an association between UA and incident or prevalent CKD, while other studies found no association^21,22^, evidencing the controversial role of UA in the incidence and progression of CKD. Furthermore, to the best of our knowledge, no review has been performed to assess the association between different UA levels (subgroups of UA levels) and the incidence and progression, contemplating all stages of CKD. Therefore, we performed a systematic review and meta-analysis of cohort studies to analyze the association of serum UA levels with the incidence and progression of CKD.

## Methods

### Protocol and register

Systematic review study conducted according to protocol registered in the International Prospective Register of Systematic Reviews (PROSPERO), under identification number CRD42020142073, respecting the recommendations of the Cochrane Collaboration, reference for the preparation and publication of systematic reviews and meta-analyses. The results were presented according to recommendations proposed by the Preferred Reporting Items for Systematic Reviews and Meta-Analyses (PRISMA)^23^.

### Search strategy

The identification and selection of the studies occurred from December 2019 to January 2020, completely independently by two researchers. The health databases consulted were: US National Library of Medicine National Institutes of Health (PubMed)/Medical Literature Analysis and Retrieval System Online (Medline), Latin American and Caribbean Center on Health Sciences Information (Lilacs) and Web of Science, main health science databases. The descriptors used in the search are indexed in the Medical Subject Headings (MeSH), which are: “Hyperuricaemia”, “Uric Acid”, “Renal Insufficiency, Chronic”, “Kidney Diseases”, “Kidney Failure, Chronic”, “Chronic Kidney Disease”. The following combinations were used: “Hyperuricaemia” or “Uric Acid” and “Renal Insufficiency, Chronic”; “Hyperuricaemia” or “Uric Acid” and “Kidney Diseases”; “Hyperuricaemia” or “Uric Acid” and “Kidney Failure, Chronic”; “Hyperuricaemia” or “Uric Acid” and “Chronic Kidney Disease”. In order to contemplate the entire scientific production, the publication period was not delimited.

### Selection of studies

After searching the databases, duplicate studies were excluded. Next, a refining was carried out to select the studies related to the theme addressed through the reading of titles and abstracts.

Studies were included if they met the following criteria: longitudinal cohort studies that evaluated the role of UA in the progression and/or incidence of CKD and had a follow-up of at least one year.

To reduce any confounding variables that may affect the association between CKD and UA, we excluded: studies with animals, children, adolescents, pregnant women and kidney transplant recipients, cross-sectional or case–control studies (to reduce the reverse causation, bias from pre-existing CKD on UA levels), studies addressing acute kidney disease and/or specific types of kidney disease (contrast-induced nephropathy or persistent post-treatment, renal failure after percutaneous coronary intervention, immunoglobulin A nephropathy, autosomal dominant polycystic kidney disease, lupus nephritis), studies on drug effectiveness, studies whose outcome was mortality and studies that assessed risk factors in general for progression of CKD (obesity dyslipidemia, diabetes, hypertension, etc.), whose focus was not to evaluate the role of uric acid.

As they were different measures, the outcomes progression and incidence of CKD were presented separately. Incidence of CKD was defined as individuals who were free of CKD at baseline (glomerular filtration rate (GFR) ≥ 60 mL/min/1.73 m^2^) but experienced a decline in GFR to less than 60 mL/min/1.73 m^2^ during follow-up. Progression was defined as decline in GFR and/or end-stage renal failure, requiring renal replacement therapy.

### Data extraction

The following data were extracted from the selected studies: author’s name, date of publication (year), study design (follow up time), characteristics of the studied population, GFR estimation equation, exposure variable, main outcome, adjustment for possible confounding factors in multivariate analyses and evidence quality analysis.

### Evidence quality analysis

The Newcastle–Ottawa Scale (NOS) was used to assess the risk of bias for cohort studies. We used three items to assess study quality: (1) selection of participants (including four domains); (2) comparability of study results (including one domain); and (3) outcome (including two domains)^24^. Each domain had a rating of “yes,” “no”, or “unclear.” If there was adequate data against a domain in the included study and met the criteria, it was classified as low risk of bias (yes). Conversely, a domain was classified as high risk of bias if adequate information was not available (no) or not enough data was available to make an assessment (unclear). “Yes” was scored as “1”, and “no” or “unclear” was scored as “0.” Scores were tallied up to calculate the final cumulative score. A study was considered high quality if the cumulative score was ≥ 4, and low quality if < 4^25^.

### Statistical analyses

For the quantitative analysis, studies with data stratified by groups according to serum UA levels (quartiles or quintiles) were selected. To evaluate the gradient, the groups with the lowest levels of UA were compared to the other groups (For quartiles: Q1 vs. Q2; Q1 vs. Q3 and Q1 vs. Q4. For quintiles: Q1 vs. Q2; Q1 vs. Q3; Q1 vs. Q4; Q1 vs. Q5).

For incidence studies, the absolute number of incident and non-incident cases in each group was used to calculate the relative risk (RR), with a 95% confidence interval and p values. For progression studies, the absolute number of cases that progressed and did not progress in each group was used to calculate the RR, with a 95% confidence interval and p values.

The effect estimates rate ratios were combined in the meta-analysis to calculate the overall risk estimate. A random-effects model was used for analysis to account for the variation of real effects across studies. Heterogeneity was quantified using the I^2^ statistic, which describes the proportion of total variation in the study estimates due to heterogeneity^26^. The degree of heterogeneity was assessed from the I^2^ statistic using the following thresholds for interpretation: (1) 0% to 30%: marginal heterogeneity; (2) 30% to 50%: moderate heterogeneity; (3) 50% to 75%: substantial heterogeneity; and (4) 75% to 100%: represents considerable heterogeneity^26^. The estimation of each study and the standard error (SE) generated a combined estimate, graphically represented by the forest plot.

Meta-regressions (univariate and multivariate) were performed to identify the causes of heterogeneity. The following variables were investigated: sex (difference in percentage of men between comparison groups), serum UA level (mean difference between comparison groups), age (mean difference between comparison groups) and sample size (mean difference between comparison groups). Initially, a univariate analysis was performed and all variables that values of p ≤ 0.200 were included in the final multivariate model. Variables with p values < 0.05 in the multivariate analysis were considered significant.

The publication bias was tested by funnel plot and Egger test. All analyses were performed using the ‘meta’ package in STATA version 14.

## Results

### Selection of studies

**Figure 1:**
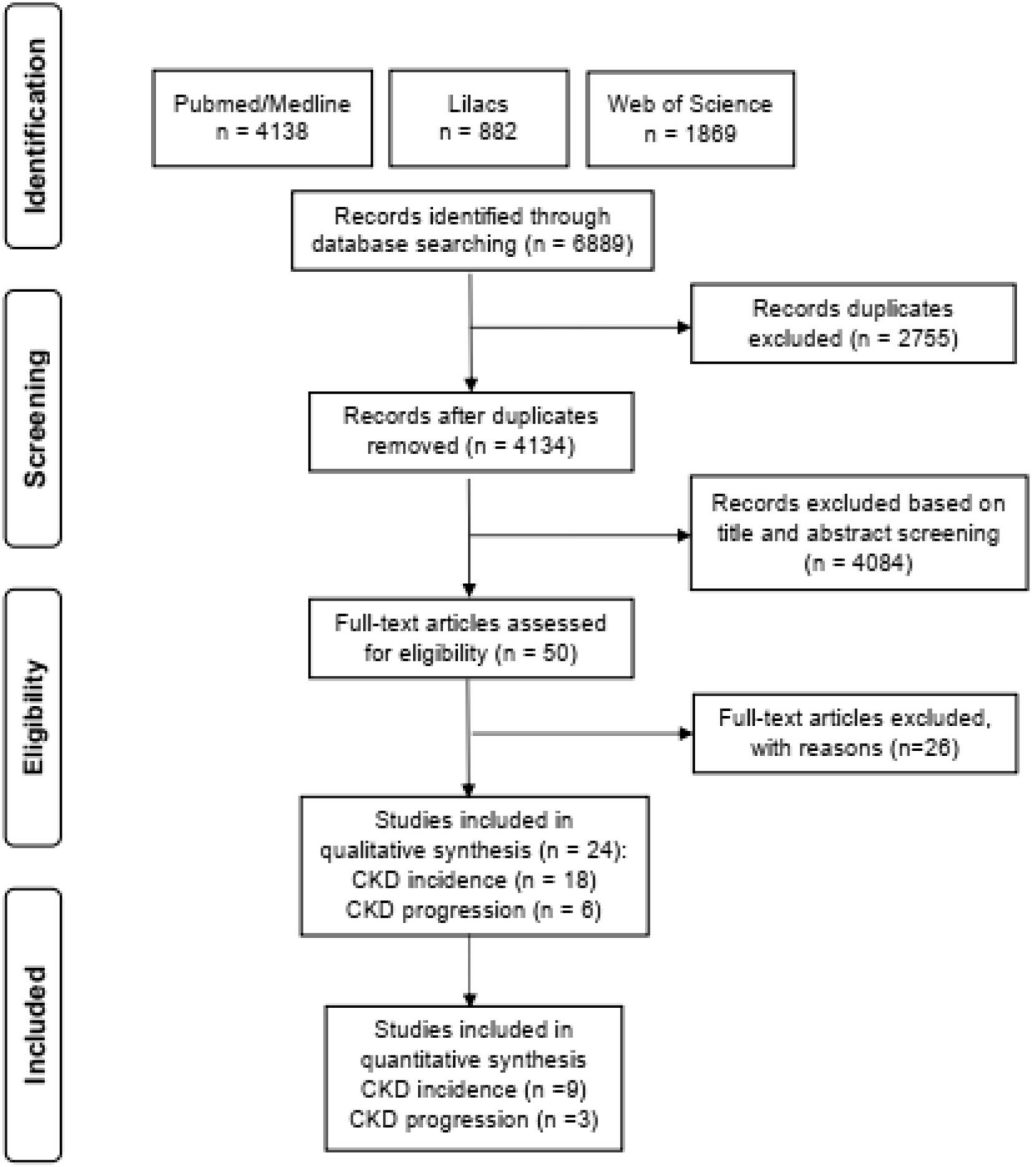
Flowchart of the process of study selection.

We identified 6889 publications at the electronic databases. After exclusion by duplicity, 4134 studies remained. In the paired selection through the screening by the titles and abstracts, 4084 more articles that did not fit our scope were eliminated. Among the 50 studies selected for full reading, we excluded 26 that did not meet the eligibility criteria. Finally, 24 studies were included and stratified into two groups: 18^6,15,16,18,20,27–39^ related to the incidence of CKD in the general population and six^5,17,21,40–42^ on CKD progression. For the quantitative analysis, nine incidence studies^6,16,28,30,32,34,35,37,39^ and three progression studies^5,17,21^ were included. Figure 1 represents the flowchart of identification and selection of studies for systematic review and meta-analysis.

### Characteristics of the studies

Tables 1 and 2 summarizes the characteristics of the 24 selected articles. All studies were longitudinal observational cohort studies, 18 prospective and six retrospective. Of the total, 14 studies were of Asian origin and 10 were conducted in Western countries between 2008 and 2019. In all, 412,238 participants were identified, 398,663 in incidence studies and 13,575 in progression studies. Regarding the confounding factors used in the adjustments of the analyzed studies, there was a diversity of variables: hypertension and/or blood pressure values; diabetes and/or related tests; dyslipidemia and/or lipidogram; use of medications (antihypertensive, diuretics, UA-lowering and lipid-lowering); proteinuria or albumin-creatinine ratio and GFR. All studies had a cumulative quality score of ≥ four and were thus considered high quality (Tables 1, 2, Supplementary table).

**Table 1:** Observational studies of the association between elevated UA level and CKD incidence.

Author (year)	Cohort design (follow-up)	N (*n* new-onset CKD) population	eGFR equation	Exposure variable	Outcome	Adjustment	Evidence quality analysis (NOS)
Kuwabara et al.^35^	Retrospective 2004–2009 (5 years)	12,578 adults non-CKD, (n = 3144 rapid eGFR decline (Q4), 30–85 years St. Luke’s International Hospital, Japan	Japanese GFR equation	Quartiles of UA	Incident CKD (eGFR < 60)	Age, gender, BMI, smoking, HTN, DM, dyslipidemia, abdominal circumference	High
Obermayr et al.^15^	Prospective (mean 7.4 ± 3.9 years)	21,475 adults non-CKD, ♀: 20–84, ♂: 20–89 years VHS Project, Áustria	MDRD	*Elevated uric acid level (≥ 9.0 mg/dl) compared with the reference group (UA < 7.0 mg/dl)	Incident CKD (eGFR < 60)	Baseline eGFR, gender, age, antihypertensive drugs, metabolic syndrome (waist circumference, HDL-C, blood glucose, triglycerides, BP)	High
Sonoda et al.^36^	Prospective (median 1694 days)	7078 adults non-CKD, (n = 568 CKD), mean age 52.8 ± 10.7 years. Health checkup program, Japan	Japanese GFR equation	*UA levels (per 1-mg/dL increase)	Incident CKD (eGFR < 60)	BMI, SBP, HDL, LDL, Hb, smoking	High
Cao et al.^37^	Prospective (mean 52.8 months)	6495 adults non-CKD, (n = 372 CKD), 35–74 years. Health Manage-ment Center of the Third Xiangya Hospital Checkup, China	Two-level CKD-EPI formula	Quartiles of UA	Incident CKD (eGFR < 60 or positive proteinuria)	Age, BMI, DM, HTN, alcohol intake, SBP, total cholesterol, eGFR, and previous use of ARBs	High
Chini et al.^38^	Retrospective 2008–2014 (mean 5.05 ± 1.05 years)	1094 adults non-CKD, (n = 44 CKD), mean age 48.7 ± 8.8 years, Eletric company's annual medical checkup, Brazil	CKD-EPI	*UA levels (per 1-mg/dL increase)	Incident CKD (eGFR < 60)	Female gender, age, DM, HTN, HDL-C, triglycerides, BMI, sedentary lifestyle, smoking	High
Kamei et al.^39^	Prospective 2008–2010 (2 years)	141,514 adults non-CKD, (n = 9169 CKD), 29–74 years (mean age 63.3 years), annual SHCG, Japan	Japanese GFR equation	Quintiles of UA	Incident CKD (eGFR < 60)	Gender, age, obesity, HTN, DM, dyslipidemia, smoking, alcohol intake, eGFR, proteinuria	High
Storhaug et al.^27^	Prospective (7 and 13 years)	2637 adults, 2215 non-CKD (n = 697), 25–74 years (mean age 57.2 years), Tromsø Study, Norway	CKD-EPI	*UA levels (per 1-mg/dL increase)	RD (ACR ≥ 1.13 mg/mmol and/or eGFR < 60)	SBP, BMI, cholesterol, current smoking; physical activity, antihypertensive drugs included diuretics, DM, myocardial infarction and/or stroke, change in SBP, starting antihypertensive treatment, cessation of smoking or becoming physically active during observation, baseline eGFR	High
Takae et al.^28^	Prospective (5 years)	2059 adults non-CKD, (n = 396), ≥ 40 years, Hisayama Study, Japan	CKD equation with a Japanese coeficient	Quartiles of UA	Incident CKD (eGFR < 60 or U-ACR ≥ 30 mg/g)	Age, sex, SBP, antihypertensive agents use, DM, HDL-C, BMI, total cholesterol, Hb, uric acid-lowering agents use, UACR, CRP, baseline eGFR, smoking, alcohol intake, regular exercise	High
Weiner et al.^16^	Prospective (mean 8.5 ± 0.9 years)	13,338 adults non-CKD, (n = 1014 CKD), mean age 57.4 ± 9.0 years, ♀ (56.6%) ARIC and CHS, USA	MDRD	Quartiles of UA	Incident CKD (eGFR decrease ≥ 15 or eGFR < 60 or SCr increase ≥ 0.4 where baseline SCr < 1.4 [♂] or < 1.2 [♀])	Age, gender, race, DM, SBP, HTN, CVD, LVH, smoking, alcohol use, education, lipids, diuretic, sAlb, Hct, baseline kidney function and cohort, diuretics	High
Zhang et al.^29^	Prospective (4 years)	1410 adults non-CKD, (n = 168 renal function decline), mean age 59.1 ± 9.4 years, 48.5% ♂, urban district of Beijing, China	modified MDRD for Chinese patients with CKD	*UA levels (per 1-mg/dL increase)	Renal function decline (baseline eGFR < 90 and ↓eGFR ≥ 20% in 4 years of follow-up; and/or ↓eGFR ≥ 20% during 4 years of follow-up and eGFR < 60 at the 2nd visit)	Age, sex, BMI, current smoking, HTN, DM, sAlb, baseline eGFR	High
Mwasongwe et al. ^30^	Prospective (median: 8.1 years)	3702 adults african american (3556 non-CKD), (n = 268 CKD) 21–94 years, mean age 55.25 ± 12.40 years, 64.5%♀, JHS,USA	CKD-EPI	Quartiles of UA	Incident CKD (eGFR < 60 with a ≥ 25% decline in eGFR between baseline and exam 3 (2009–2013)	Age, sex, BMI, eGFR, gout medications, loop diuretics, thiazide diuretics, potassium-sparing diuretics, antihyperlipidemics, DM, total cholesterol, CRP, UACR	High
Ben-Dov e Karc^31^	Prospective (24–28 years)	2449 adults non-CKD (1470 ♂ [n = 87 CKD] 979 ♀ [n = 22 CKD]), 35–78 years, Jerusalem LRC	MDRD or CKD-EPI	*Quintiles of UA levels: Q5 (♂ > 6.5 mg/dL or ♀ > 5.3 mg/dL) Q5 versus Q1-4 UA	Incident CKD (defined by hospital discharge records)	Glucose, smoking, globulins, birth origin (Israel, Europe, Asia, North Africa), DM medication, protein and alcohol consumption, SBP DBP, Hct age, secular education level (years), protein and alcohol consumption, diabetes medication status (DM med), BMI, triceps skinfold thickness, systolic and diastolic blood pressure, hematocrit, creatinine, globulins, serum AST, thyroxine, bilirubin, fasting glucose (ln), total cholesterol, triglycerides (ln), LDL-C, HDL-C and very low-density lipoprotein cholesterol, urine protein (stick)	High
Chou et al.^6^	Prospective 2002–2007 (mean 5.18 years)	3605 adults non-CKD, (n = 233 CKD) 39.52 ± 14.63 years; 45.6% ♂ TwSHHH I-II, Taiwan	CKD-EPI	Persistently (high vs. low) UA level (4 groups corresponding to quartiles)	Incident CKD (eGFR < 60 or proteinuria ≥ 2 +)	Sex, age, HTN status, BMI, total cholesterol, triglycerides, FPG, and eGFR	High
Kuo et al.^18^	Retrospective 1996–2008 (12 years)	63,785 adults non-CKD (n = 7964 CKD), mean age 50.0 ± 14.9 years, Chang Gung Medical Foundation, Taiwan	MDRD	*Hyperuricaemia (♂ > 7.7, ♀ > 6.6) HU versus NU group	Incident CKD (eGFR < 60)	Age, sex, DM, HTN, baseline eGFR, hypercholesterolemia, azotemia, hyperglycemia	High
Mok et al.^32^	Prospective 1994–2004 (10.2-year)	14,939 adults non-CKD, (8685 ♂ [n = 438 CKD] 6254♀ [n = 328 CKD]), 20–84 years, Severance Health Promotion Center, Korea	MDRD	Quartiles of UA	Incident CKD (eGFR < 60)	Age, smoking status, alcohol consumption, exercise, BMI, HTN, DM, dyslipidemia (cholesterol)	High
Bellomo et al.^33^	Prospective (5 years)	900 adults non-CKD (153 ♂ [n = 10 CKD*] 747♀ [n = 1 CKD*]), 20–65 years, blood donors at a hospital transfusion center, Italy	CKD-EPI	*UA levels (per 1-mg/dL increase)	eGFR decrease > 10	Age, sex, BMI, blood glucose level, mean BP, UACR, total cholesterol level, baseline eGFR, triglycerides	High
Wang et al.^35^	Retrospective 1997 and 2004 (mean 3.5 years)	94 422 adults non-CKD (n = 3683 CKD), ≥ 20 years (age range 20.00–93.72 years), 50.4% ♂, MJLPD, Taiwan	MDRD and CKD-EPI	*UA levels (per 1-mg/dL increase)	Incident CKD (eGFR < 60)	Age, sex, BMI, education, alcohol, smoking, exercise, triglyceride, total cholesterol, LDL-c, HDL-c, sAlb, CRP, GGT, SUN, eGFR, proteinuria, medical/family history (HTN, DM), medications (allopurinol, antihyperlipidemic drug, Chinese herbal medicine), SBP, DBP, FPG	High
Ye et al.^34^	Retrospective 2011–2016 (6 years)	5183 adults non-CKD (3176 ♂ [n = 139 CKD] 2007♀ [n = 88 CKD]), 25–85 years, Zhejiang Province People’s Hospital check-up, China	Modified MDRD for Chinese patients with CKD	Quartiles of UA	Incident CKD (eGFR < 60)	Age, sex, BMI, SBP, DBP, Total cholesterol, baseline eGFR, FPG, Hyperuricaemia, HTN, DM	High

N: sample size; n: number of outcomes ; eGFR: estimated glomerular filtration rate (em ml/min/1.73 m^2^); OR: odds ratio; HR: Hazard ratio; CI: confidence interval; CKD: chronic kidney disease; UA: serum uric acid level; BMI: body mass index; HTN: hypertension; DM: diabetes mellitus; ♂: male; ♀: female; VHS Project: Vienna Health Screening Project; MDRD: Modification of Diet in Renal Disease; UA: uric acid; HDL-C: high-density lipoprotein cholesterol; BP: blood pressure; SBP: systolic blood pressure; LDL-C: low-density lipoprotein cholesterol; Hb: hemoglobin; CKD-EPI: chronic kidney disease epidemiology; Q4: 4th quartile of uric acid level; vs: versus; Q1: 1st quartile of uric acid level; SHCG Specific Health Check and Guidance; RD: renal dysfunction; ACR: albumin-creatinine ratio; UACR: urine albumin-creatinine ratio; CRP: C-reactive protein; ARIC: Atherosclerosis Risk in the Community; CHS: Cardiovascular Health Study; USA: United States of America; SCr: serum creatinine; CVD: cardiovascular disease; LVH: left ventricular hypertrophy; ; sAlb: serum albumin; Hct: hematocrit; JHS: Jackson Heart Study; Jerusalem LRC: Jerusalem Lipid Research Clinic; Q5: 5th quintile of uric acid level; DBP: diastolic blood pressure; Hct: hematocrit; AST: alanine aminotransferase; TwSHHH: Taiwanese Survey on Prevalence of Hypertension, Hyperglycemia, and Hyperlipidemia; FPG: fasting plasma glucose; HU: hyperuricaemic; NU: normouricemic; MJLPD: Taiwan MJ Longitudinal health-checkup-based Population Database; GGT: gamma-glutamyl transpeptidase; SUN: serum urea nitrogen.

*Exposure variable—did not present enough data for the meta-analysis.

**Table 2:** Observational studies of the association between UA level and CKD progression.

Author (year)	Cohort design (follow-up)	N (*n* cases), Population	eGFR equation	Exposure variable	Outcome	Adjustment	Evidence quality analysis (NOS)
Hsieh et al.^17^	Retrospective (median 3.03 years)	2408 adults CKD stages 3–5 (n = 652 RRT), mean age: 65.7 ± 12.6 years, 56.9% ♂ CKD care program, CCH, Taiwan	MDRD	Quartiles of UA	RRT	Gender, age, BMI, DM, HTN, CVD, gout, HbA1C, cholesterol, triglyceride, BUN, eGFR, GPT, sAlb, Ca x P, WBC count, Hb, proteinuria, diuretics, hypouricemic agents, erythropoiesis stimulating agents, ACEi, ARBs	High
Liu et al.^21^	Prospective (median 2.8 years)	3303 adults CKD stages 3–5, (n = 1080 RRT), mean age: 63.5 ± 13.5 years, 57.8% ♂ ICKD, Taiwan	MDRD	Quartiles of UA	RRT	Age, sex, CVD, mean BP, BMI, HbA1C, cholesterol, smoking, CRP, eGFR, proteinuria, sAlb, Hb, bicarbonate, calcium, phosphate, ACEi, ARB, diuretics, gout, hypouricemic agent use	High
Nacak et al.^40^	Prospective (median 14.9 months)	131 adults CKD stages IV-V (n = 71 RRT), ≥ 18 years (mean age: 63.6 ± 14.6 years),68,7% ♂ PREPARE-2 study 2004–2011, Netherlands	MDRD	*Baseline UA (per 1 mg/dL increase)	Time to start of RRT (peritoneal dialysis or hemodialysis)	Age, sex, ethnicity, PKD, BMI, CVD, HTN, DM, protein restricted diet, SBP, LDL, cholesterol, proteinuria, diuretics, allopurinol	High
Nacak et al.^40^	Prospective (median 28 months)	2466 adults, (n = 530 RRT), ≥ 18 years, mean age: 69.0 ± 13.6 years, and 65% ♂.SRR-CKD, 2005–2011, Sweden	MDRD	*Baseline UA (per 1 mg/dL increase)	Time to start of RRT	Age, sex, BMI, protein-restricted diet, diuretics, lipid-lowering medication, MAP, PRD, allopurinol use, DM, arrhythmia, CVD, IHD, HTN, pulmonary disease and CHF	High
Sturm et al.^42^	Prospective (median 53 months)	177 adults CKD stages I-V (n = 65 CKD progression/n = 29 RRT), 18–65 years, mean age: 46.4 ± 12.2, 67% ♂ MMKD Study, Germany and Austria	Iohexol clearance technique	*Baseline UA (per 1 mg/dL increase)	CKD progression (doubling of baseline SCr and/or ESRD/RRT)	Sex, age, eGFR, proteinuria	High
Tsai et al.^5^	Prospective (median 31.6 months)	5090 adults CKD stages III-V (n = 948 ESRD) 20–90 years, median age: 67.2 years (IQR: 56.8–75.9), 59.4% ♂ CMUH pre-ESRD program, China	MDRD	Quartiles of UA	CKD progression (ESRD)	Age, sex, BMI, smoking, alcohol intake, education, DM, HTN, CVD, primary CKD, baseline medication (including pentoxifylline, dipyridamole, anti-platelet agents, allopurinol, febuxostat, nezbromarone, colchicine, sulfinpyrazone ACEIs, ARBs, trichlorethiazide, furosemide and other diuretics including spironolactone, amizide and indapamide), baseline eGFR, baseline UA, eGFR trajectory	High

N: sample size; n: number of outcome cases; eGFR: estimated glomerular filtration rate (em ml/min/1.73 m^YY^); HR: Hazard ratio; CI: confidence interval; CKD: chronic kidney disease; ♂: male; CCH: Changhua Christian Hospital; MDRD: Modification of Diet in Renal Disease; UA: serum uric acid level; RRT: renal replacement therapy; BMI: body mass index; DM: diabetes mellitus; HTN: hypertension; CVD: cardiovascular disease; HbA1C: glycated haemoglobina; BUN: blood urea nitrogen; GPT: glutamic-pyruvic transaminase; sAlb: serum albumin; WBC, white blood cell; Hb: hemoglobin; ACEi: angiotensin converting enzyme inhibitors; ARBs: angiotensin II receptor blockers; ICKD: Integrated CKD care program Kaohsiung for delaying Dialysis; BP: blood pressure; CRP: C-reactive protein; PREPARE-2: PRE-dialysis PAtient REcord-2SRR-CKD; PKD: Polycystic kidney disease; SBP: systolic blood pressure; LDL-C: low-density lipoprotein cholesterol; SRR-CKD: Swedish Renal Registry–Chronic Kidney Disease; MAP: mean arterial pressure; PRD: primary renal disease; IHD: interstitial heart disease; CHF: chronic heart failure; MMKD: Mild to Moderate Kidney Disease Study; SCr: serum creatinine; ESRD: end-stage renal disease; CMUH: China Medical University Hospital.

*Exposure variable—did not present enough data for the meta-analysis.

### Synthesis of the results

#### UA levels and CKD incidence

**Figure 2:**
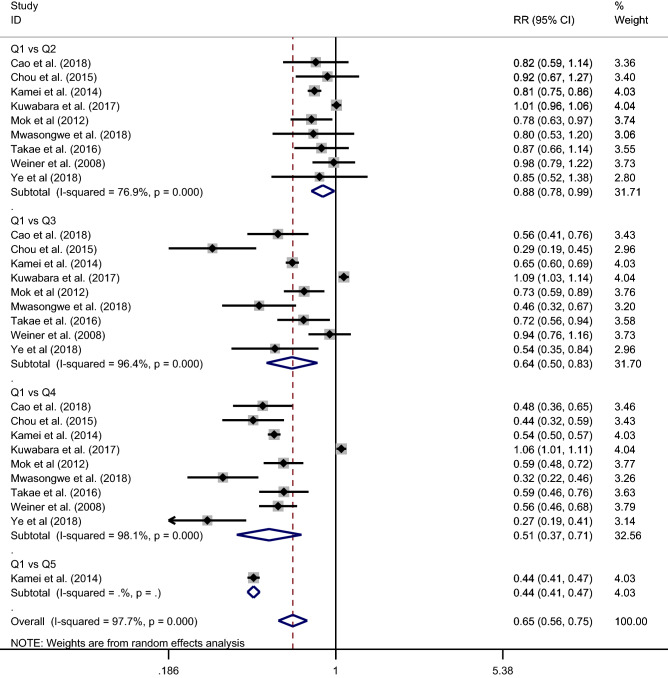
Forest-plot of the meta-analysis of cohort studies that investigated the association of UA levels and CKD incidence.

An inverse relationship was observed between UA levels and protection for the CKD incidence (Fig. 2). Lower UA levels were protective for the risk of CKD incidence (RR 0.65 [95% CI 0.56–0.75]). With increasing quartiles or quintiles, protection for the CKD incidence decreased: Q1 versus Q2 (RR 0.88 [95% CI 0.78–0.99]), Q1 versus Q3 (RR 0.64 [95% CI 0.50–0.83]), Q1 versus Q4 (RR 0.51 [95% CI 0.37–0.71]), Q1 versus Q5 (RR 0.44 [95% CI 0.41–0.47]). In other words, the risk of CKD incidence increased with increasing levels of UA, and overall, the risk for incidence was 1.54.

The meta-analysis showed considerable heterogeneity by the I^2^ statistic (I^2^ = 97.7%). Regarding meta-regression (Table 3), in the univariate analysis, the age difference between the groups was the only variable with a *p* value < 0.200, therefore, the multivariate analysis was not necessary. We conclude that increasing the age difference between the groups increased the risk ratio between UA and the incidence of CKD (Coeff β 1.19 [95% CI 1.12–1.26]).

**Table 3:** Meta-regression analysis to explore the effects of the study characteristics on CKD incidence.

Variables	Univariate	
	Coeff. Β (95% CI)	*p*
UA level	1.36 (0.44–4.17)	0.509
Age	1.19 (1.12–1.26)	< 0.001
Sex	0.99 (0.98–1.00)	0.391
Sample size	1.00 (0.99–1.00)	0.466

**Figure 3:**
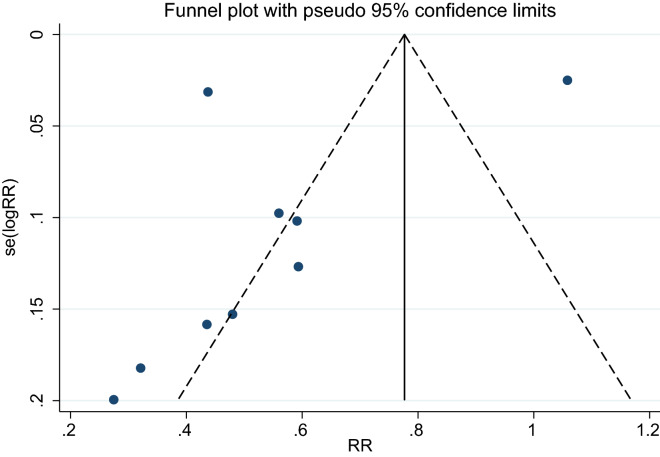
Funnel plot of the studies that assessed the association between UA levels and CKD incidence.

Funnel plot analysis qualitatively showed an asymmetric shape (Fig. 3), indicating the possibility of publication bias for the association between UA levels and CKD incidence. However, the Egger test showed no indication of publication bias (*p* = 0.249).

#### UA levels and CKD progression

**Figure 4:**
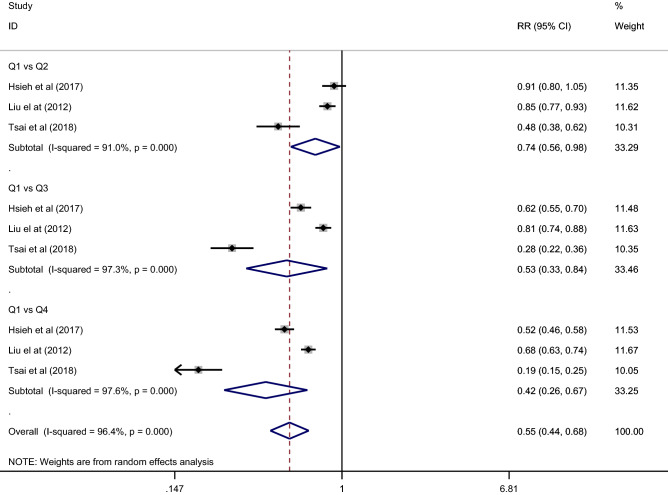
Forest-plot of the meta-analysis of cohort studies that investigated the association of UA levels in patients with CKD in stages III-IV and their progression to terminal CKD or early initiation of renal replacement therapy.

An inverse relationship was observed between UA levels and protection from CKD progression (Fig. 4). Lower UA levels were protective for the risk of CKD progression (RR 0.55 [95% CI = 0.44–0.68]). With increasing quartiles or quintiles, protection for CKD progression decreased: Q1 versus Q2 (RR 0.74 [95% CI = 0.56–0.98]), Q1 versus Q3 (RR 0.53 [95% CI = 0.33–0.84]), Q1 versus Q4 (RR 0.42 [95% CI = 0.26–0.67]). In other words, the risk of CKD progression increased with increasing UA levels, and overall, the risk for progression was 1.81.

The meta-analysis showed considerable heterogeneity by the I^2^ statistic (I^2^ = 96.4%). Regarding meta-regression (Table 4), in the multivariate analysis no variable was significantly associated with the risk relationship between UA and CKD progression (Table 4).

**Table 4:** Meta-regression analysis to explore the effects of the study characteristics on CKD progression.

	Univariate		Multivariate	
	Coeff. β (95% CI)	*p*	Coeff. Β (95% CI)	*p*
UA level	1.32 (0.74–2.33)	0.288	–	
Age	0.54 (0.25–1.17)	0.103	0.76 (0.21–2.70)	0.619
Sex	1.13 (0.98–1.30)	0.077	1.08 (0.85–1.39)	0.423
Sample size	0.99 (0.99–1.00)	0.422	–	

Funnel plot analysis and Egger's test were not performed for CKD progression because few studies were selected.

## Discussion

The present study found a gradient relationship between UA levels and CKD, with lower UA levels were protective for the risk of CKD incidence and progression. In addition, increasing the age difference between groups (with different levels of UA) increased the risk between UA and incidence of CKD.

UA levels are an independent predictor of the development of CKD and its progression. Some studies have shown that the prevalence of hyperuricaemia gradually increases with the decrease in renal function, being 10 times higher in stages 3–5 than in stage 1^43^. This relationship highlights a potential benefit of UA screening in different phases of CKD^44^.

A retrospective cohort involving 13,133 healthy adults (without hypertension, diabetes, obesity or CKD) showed that increasing UA levels doubled the risk of incident CKD^45^. Similarly, a Japanese retrospective cohort with 5,507 adults followed-up for an average period of 4.6 years found a positive association of hyperuricaemia (≥ 7 mg/dl) with the incidence of CKD (adjusted HR = 1.58 [95% CI = 1.21–2.07]), but with no effect on its progression (HR = 1.08 [95% CI = 0.73–1.59])^46^.

Jalal^47^ made a critical review of observational and experimental studies on the potential effect of UA reduction therapy on the prevention of the incidence and progression of CKD and concluded that UA participates in inflammation and evolution of CKD. The authors acknowledge, however, that such conclusions are controversial because they are based on small studies and without placebo group.

Thus, two recently published double-blind, placebo-controlled, multicenter randomized trial^48,49^ concluded the use of allopurinol was not beneficial to prevent the progression of pre-existing kidney disease, with no statistical difference between using or not using the UA-lowering agents. On the other hand, a recent systematic review and meta-analysis of 11 randomized clinical trials, including 4,277 participants with CKD, suggested that UA-lowering therapy preserves GFR so that xanthine oxidoreductase inhibitors could improve renal outcomes^50^. This finding may indicate a different role for UA in the pathophysiology of CKD in addition to a nitrogen slag.

The definition of the role of UA is still complex, especially due to the coexistence of risk factors such as hypertension, chronic inflammation and pathophysiological peculiarities of CKD. Recent studies have shown that soluble UA exhibits a behavioral duality acting as pro-oxidant within the cell and antioxidant in the extracellular environment^10,51,52^.

In relation to the pro-oxidant effect, the UA stimulates the generation of reactive oxygen species culminating in oxidative stress and mitochondrial damage, endothelial dysfunction with activation of the renal-angiotensin-aldosterone system, reduction of nitric oxide bioavailability and afferent arteriolopathy^53–55^. Furthermore, UA induces the activation of dependent or independent pathways of NLRP3 inflamassome with release of pro-inflammatory cytokines (IL1β and IL18), necro-inflammation and interstitial fibrosis^56^. Such pathophysiological mechanisms may justify the role of UA in the incidence and progression of CKD.

There were several strengths to this systematic review: 1. it is one of the first studies to gather evidence from observational studies in the search for an association of UA levels, not only with the incidence of CKD, but also with its progression to terminal stages, thus contemplating all stages of CKD; 2. the literature search included several large databases with the search criteria designed to identify as many relevant articles as possible; 3. a proportion of the study selection, data extraction and quality assessment were conducted in duplicate by separate reviewers to reduce reporting bias; 4. all studies were classified as high quality and encourages future researches related to the use of UA reduction therapies for preventing CKD in at-risk populations.

There are some limitations to our study that need to be taken into consideration. First, the studies presented heterogeneous sample sizes, which was minimized with the use of appropriate statistical tests in the meta-analysis. Secondly, different definitions were observed for the final outcome (GFR < 60, decline in GFR, rapid decline of GFR) and for the independent variable (UA levels). Subgroup analysis based on the UA levels (quartiles or quintiles) was conducted to assess whether the presentation of exposure contributed to differences in study results. This analysis showed similar results regardless of the method of presenting the exposure. Thirdly, different forms of GFR estimation (MDRD, CKD-EPI, Cockcroft-Gault, Japanese equation, equation based on insulin clearance, iohexol technique) were adopted. Finally, there was some heterogeneity among risk estimates from the included studies, possibly due to some bias at the study level. However, the variables included in the meta-regression were not sufficient to explain the heterogeneity between studies. Other important variables that interfere in the UA metabolism could not be included, as they were not present in all studies, such as gout, basal GFR, diet components, use of UA-lowering agents.

In conclusion, the present study provides evidence that lower UA levels were protective for the risk of CKD incidence and progression. Increasing the age difference between the groups increased the risk between UA and the incidence of CKD.

## Supplementary Information

Supplementary Information 1.

Supplementary Information 2.
