# Supplementary material for: A systematic review and meta-analysis of the association between uric acid levels and chronic kidney disease
Source: Sci Rep. 2022 Apr 15;12:6251. doi: 10.1038/s41598-022-10118-x (PMC9012819; doi:10.1038/s41598-022-10118-x)
Supplement: Supplementary file 2 — Supplementary Information 2. [file 41598_2022_10118_MOESM2_ESM.docx]

**S1 Table. Risk of Bias summary for individual studies**

|  | **Selection of participants** | | | | **Comparability** | **Outcome** | |  |
| --- | --- | --- | --- | --- | --- | --- | --- | --- |
| **Study** | **Representativeness of the exposed cohort** | **Selection of the non exposed cohort** | **Ascertainment of exposure** | **Demonstration that outcome of interest was not present at start of study** | **Comparability of cohorts on the basis of the design or analysis** | **Assessment of outcome** | **Follow-up long enough for outcomes to occur** | **Quality**  **(NOS)** |
| Kuwabara et al. (2017) | 1 | 1 | 1 | 1 | 1 | 1 | 1 | High |
| Obermayr et al. (2008) | 1 | 1 | 1 | 1 | 1 | 1 | 1 | High |
| Sonoda et al. (2011) | 1 | 1 | 1 | 1 | 1 | 1 | 1 | High |
| Cao et al. (2018) | 1 | 1 | 1 | 1 | 1 | 1 | 1 | High |
| Chini et al. (2017) | 1 | 1 | 1 | 1 | 1 | 1 | 1 | High |
| Kamei et al. (2014) | 1 | 1 | 1 | 1 | 1 | 1 | 1 | High |
| Storhaug et al. (2015) | 1 | 1 | 1 | 1 | 1 | 1 | 1 | High |
| Takae et al. (2016) | 1 | 1 | 1 | 1 | 1 | 1 | 1 | High |
| Weiner et al. (2008) | 1 | 1 | 1 | 1 | 1 | 1 | 1 | High |
| Zhang et al. (2012) | 1 | 1 | 1 | 1 | 1 | 1 | 1 | High |
| Mwasongwe et al. (2018) | 1 | 1 | 1 | 1 | 1 | 1 | 1 | High |
| Ben-Dov e Karc (2011) | 1 | 1 | 1 | 1 | 1 | 1 | 1 | High |
| Chou et al (2015) | 1 | 1 | 1 | 1 | 1 | 1 | 1 | High |
| Kuo et al (2011) | 1 | 1 | 1 | 1 | 1 | 1 | 1 | High |
| Mok et al (2012) | 1 | 1 | 1 | 1 | 1 | 1 | 1 | High |
| Bellomo et al (2010) | 1 | 1 | 1 | 1 | 1 | 1 | 1 | High |
| Wang et al (2011) | 1 | 1 | 1 | 1 | 1 | 1 | 1 | High |
| Ye et al (2018) | 1 | 1 | 1 | 1 | 1 | 1 | 1 | High |
| Hsieh et al (2017) | 1 | 1 | 1 | 1 | 1 | 1 | 1 | High |
| Liu el at (2012) | 1 | 1 | 1 | 1 | 1 | 1 | 1 | High |
| Nacak et al (2014) | 0 | 1 | 1 | 1 | 1 | 1 | 1 | High |
| Nacak et al (2015) | 1 | 1 | 1 | 1 | 1 | 1 | 1 | High |
| Sturm et al (2008) | 0 | 1 | 1 | 1 | 0 | 1 | 1 | High |
| Tsai et al (2018) | 1 | 1 | 1 | 1 | 1 | 1 | 1 | High |

Note: NOS = Newcastle-Ottawa Scale, 1 = the study satisfied the criteria, 0 = the study did not satisfy the criteria or it was unclear whether the study satisfied the crietria.
